# Thoracic, abdominal and musculoskeletal involvement in Erdheim-Chester disease: CT, MR and PET imaging findings

**DOI:** 10.1007/s13244-014-0331-7

**Published:** 2014-07-14

**Authors:** Célia Antunes, Bruno Graça, Paulo Donato

**Affiliations:** Centro Hospitalar e Universitário de Coimbra, Praceta Prof. Mota Pinto, 3000-075 Coimbra, Portugal

**Keywords:** Erdheim-Chester disease, Osteoclerosis, Hairy kidney sign, Coated aorta

## Abstract

**Background:**

Erdheim-Chester disease (ECD) is a rare, non-Langerhans cell histiocytosis with characteristic radiological and histological features. This entity is defined by a mononuclear infiltrate consisting of lipid-laden, foamy histiocytes that stain positively for CD68 and negatively for CD1a. Osseous involvement is constant and characteristic. Extra-osseous lesions may affect the retroperitoneum, lungs, skin, heart, brain and orbits.

**Methods:**

Both radiography and technetium-99m bone scintigraphy may reveal osteosclerosis of the long bones, which is a typical finding in ECD. For visceral involvement, computed tomography (CT) is most useful, while magnetic resonance (MR) imaging is more sensitive for cardiovascular lesions; 2-[fluorine-18] fluoro-2-deoxy-d-glucose (FDG) positron emission tomography (PET)/CT scanning is useful in assessing the extension of ECD lesions.

**Results:**

The prognosis is extremely variable and is often worse when there is cardiovascular system involvement. Diagnosis is based on the combination of radiographic, CT, MR imaging and nuclear medicine features and a nearly pathognomonic immunohistochemical profile.

**Conclusion:**

The aims of this work are to perform a systematic review of Erdheim-Chester disease as seen on imaging of the chest, abdomen and musculoskeletal system and to discuss the diagnostic workup and differential diagnoses according to the imaging presentation.

*Teaching points*

*• Bone involvement is usually present in patients, and the imaging findings are pathognomonic of ECD.*

*• The circumferential periaortic infiltration may extend to its branches, sometimes becoming symptomatic.*

*• Cardiac involvement—the pericardium, right atrium and auriculoventricular sulcus—worsens its prognosis.*

*• Perirenal infiltration extending to the proximal ureter is highly suggestive of this disease.*

## Introduction

ECD is a rare, non-familiar disorder, first described by Jakob Erdheim and William Chester in 1930 as "lipid granulomatosis” [1]. By January 2011, approximately 400 distinct cases had been reported in the medical literature [2].

This disease usually occurs in middle-aged to older patients, with some possible cases described in the paediatric population, and has a slight male predominance [3–5]. Its aetiology and pathogenesis remain unknown because of the rarity of the disease [6], but a hereditary origin or infectious process has been excluded [4]. Recently, several researchers have demonstrated that an abnormal increase in the Th1 immune response, producing several proinflammatory cytokines, namely interferon-a, interleukin-12 and monocyte chemotactic protein-1, is probably responsible for the recruitment and activation of the histiocytes in the tissues, ruling out the hypothesis of the monoclonal proliferation of these cells [4].

The clinical manifestations in ECD are nonspecific and depend on the affected organ being asymptomatic, clinically indolent or sometimes life threatening [4].

Although recent advances in treatment have decreased the morbidity, the prognosis is still quite poor and the majority of patients die within 3 years from renal, cardiovascular, pulmonary or central neurological complications [7].

In this article, we performed a systematic review of Erdheim-Chester disease as seen on computed tomography (CT), magnetic resonance (MR) imaging or 2-[fluorine-18] fluoro-2-deoxy-d-glucose (FDG) positron emission tomography (PET)/CT imaging of the chest, abdomen and musculoskeletal system. We also discussed the diagnostic workup and differential diagnosis based on imaging findings.

### Bone

Bone involvement is almost universal in ECD (96 % of cases) [4, 5], and more than 50 % of cases have at least one associated extraskeletal involvement [5], namely the kidney, skin, central nervous system or heart. Patients may have bone pain, frequently juxta-articularly at the knees and ankles.

The imaging findings are generally typical and considered pathognomonic. The disease essentially affects the long bones and only rarely the axial skeleton [7, 4].

**Fig. 1:**
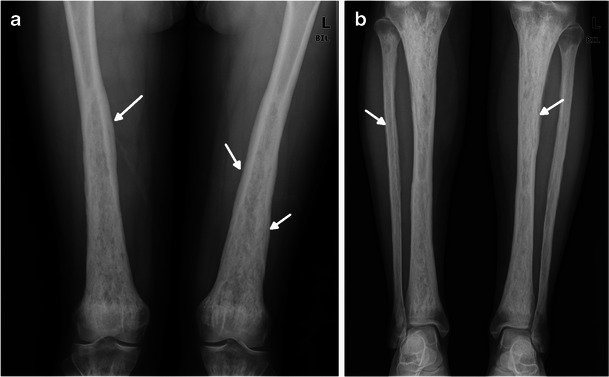
**a** AP radiographs of the distal femora in a 39-year-old female. Bone involvement is bilateral and symmetric with diaphyseal and metaphyseal heterogeneous osteosclerosis. Distal femora are enlarged, and periostitis is visible as a wavy contour (arrows) of the outer cortex. Corticomedullary margins in the diaphysis are blurred. **b** AP radiograph of two legs in the same patient. Both tibiae and peronei are involved. Corticomedullary margins in the diaphysis are blurred, and the marrow cavity is obliterated. Periostitis is also present (arrows)

The lower extremities are more commonly affected, and the disease manifests as a bilateral and symmetric cortical sclerosis associated with a homogeneous or heterogeneous sclerosis of the cancellous bone at the diaphyseal and metaphyseal regions, classically sparing the epiphyses (Fig. 1) [5]. Rarely, these alterations may be associated with periostitis and endosteal thickening (Fig. 1) [6, 8, 9].

**Fig. 2:**
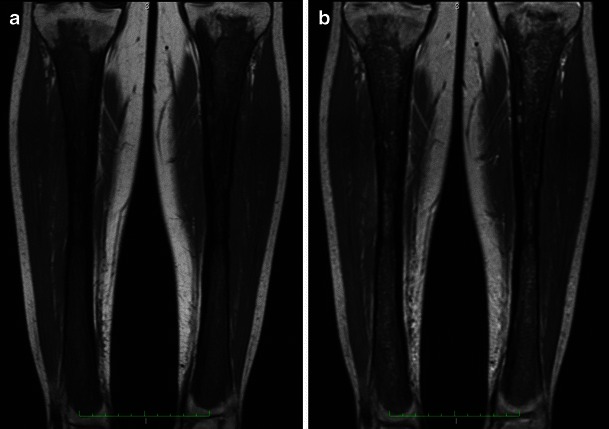
**a** Coronal T1-weighted spin-echo and **b** coronal T2-weghted spin-echo MR images of both legs in a 38-year-old female. There is symmetric low signal intensity of the diaphyses and metaphyses, sparing the epiphysis

On MR, skeletal involvement consists of extensive replacement of the fatty marrow by low signal on T1WI, heterogeneous signal on T2WI/STIR and enhancement after gadolinium injection (Fig. 2). MRI is useful to evaluate the extent of medullary bone disease and diagnose the presence of associated osteonecrosis [9, 10].

**Fig. 3:**
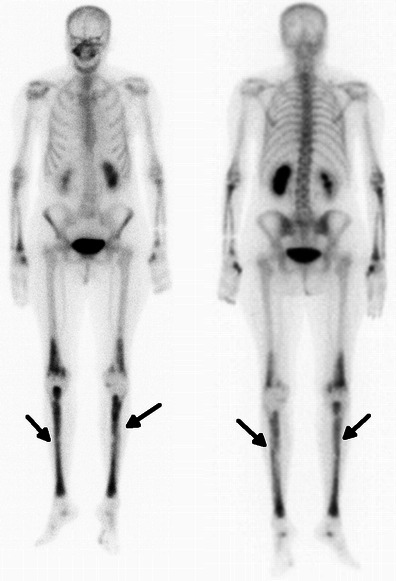
Coronal 99mTc bone scan images in a 71-year-old female. There is elevated metabolic activity in the long bones of the lower limbs, sparing the epiphysis, more exuberant in tibiae (arrows)

On technetium-99m bone scintigraphy, ECD also has pathognomonic features, namely the intense symmetric activity of the appendicular skeleton without reaching the epiphyseal regions (Fig. 3) [9].

**Fig. 4:**
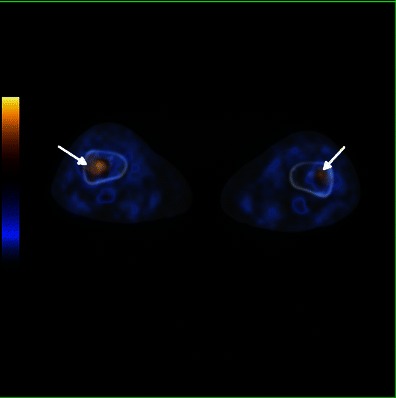
Axial FDG-PET/CT fusion images in a 71-year-old female showing elevated metabolic activity and medullary sclerosis in the long bones of the distal lower limbs (arrows)

FDG PET/CT scan reveals typical bilateral and symmetric uptake of FDG in the long bones, similar to that observed with radiography and 99mTc bone scintigraphy (Fig. 4). This imaging technique has the advantage of performing an accurate evaluation of the extent of both the skeletal and extraskeletal disease [11].

Osteosclerosis is the most common manifestation of this disease, but rarely there is a mixed pattern with lytic and sclerotic lesions, hindering the differential diagnosis with Langerhans cell histiocytosis (LCH) [4, 5] and Paget’s disease. Nevertheless, in LCH, the presence of sharply defined lytic lesions in the axial skeleton, namely in the spine, skull and mandible [28], is characteristic, and in Paget’s disease, areas beyond the axial skeleton are quite often affected; the involvement of the appendicular skeleton is often asymmetric [29].

Taking into account the age group and the sclerotic bone changes, myelofibrosis, renal osteodystrophy, osteoblastic metastases and chronic focal osteomyelitis are acquired syndromes that may mimick ECD radiographically [4, 29], thus being very important in the clinical and laboratory test correlation for making the correct diagnosis.

Other differential diagnoses of bone disease include hereditary sclerosing bone disease, namely progressive diaphyseal dysplasia [29], characterised by endosteal and periosteal thickening and narrowing of the marrow cavity, and intramedullary osteosclerosis [29], which shows unilateral or bilateral asymmetric endosteal thickening confined to the diaphysis of the long bones. However, these are usually detected in young people.

### Renal and perirenal involvement

The kidneys and retroperitoneum are involved in approximately 68 % of cases [4], being probably the most common extraosseous localisation.

Patients are mostly asymptomatic and do not present alterations in the routine blood test [7].

**Fig. 5:**
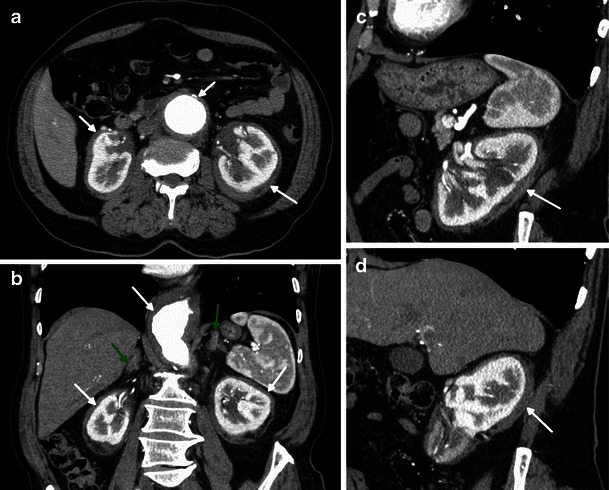
Reformatted axial (**a**), coronal (**b**) and sagittal (**c**, **d**) enhanced CT images in a 73-year-old female showing bilateral and symmetric perirenal infiltration (arrow) and bilateral adrenal thickening (green arrow). Note also the periaortic concentric soft tissue infiltration (arrow) and aortic aneurysm

**Fig. 6:**
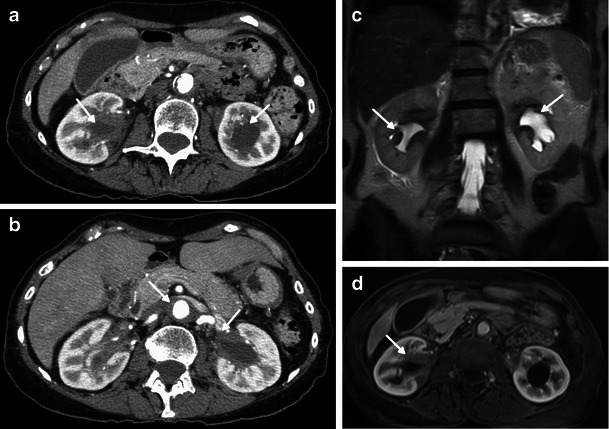
Axial enhanced CT images on a lower (**a**) and higher (**b**) abdominal level and coronal HASTE (**c**) and axial T1 post-gadolinium (**d**) MRI images in a 71-year-old female showing bilateral and symmetric infiltration of the renal sinuses, with mild hydronephrosis (arrows). Note also the periaortic concentric soft tissue infiltration (arrow)

On sectional imaging, the hairy kidney sign—a symmetric and bilateral irregular soft-tissue infiltration of both the perirenal and posterior pararenal space—is highly suggestive of this condition (Fig. 5) [12]. These infiltrations can extend to the renal sinuses and proximal ureters, causing pyelocaliceal dilatation and consequently abdominal pain and chronic renal insufficiency (Fig. 6) [13].

On CT, this manifests as a hypoattenuated and homogeneous band, with spiculated contours and weak contrast enhancement (Figs. 5 and 6). On MR imaging, it is isointense to muscle on T1- and T2-weighted sequences, with a slight and homogeneous enhancement after gadolinium injection (Fig. 6) [14].

The major differential diagnosis is retroperitoneal fibrosis, either primary or secondary, because all these diseases produce hydronephrosis by encasement of the collecting system. In ECD, the renal pelvis and proximal ureter are the portions generally obstructed by the retroperitoneal infiltrative tissue, whereas in retroperitoneal fibrosis, the distal pelvic ureters are more frequently infiltrated and also medially retracted. On the other hand, contrary to ECD, retroperitoneal fibrosis does not involve the perirenal space and spares the posterior aortic wall, but it may compress the inferior vena cava [30].

### Adrenal fossa involvement

**Fig. 7:**
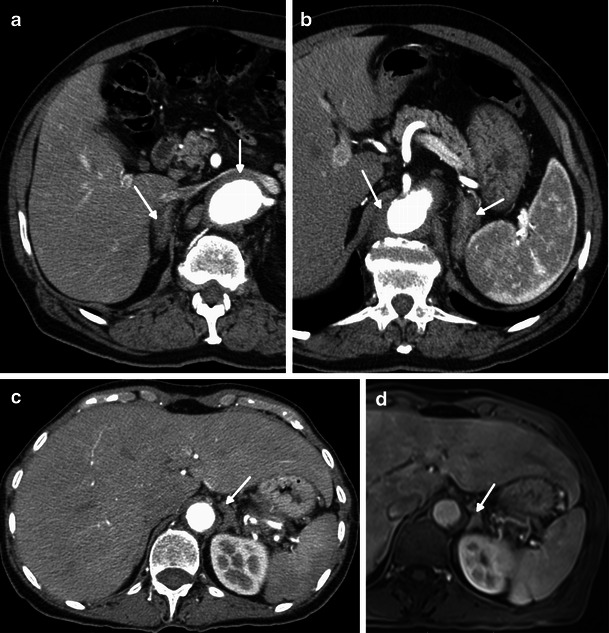
Axial CT-enhanced images in a 73-year-old male showing bilateral and diffuse thickening of the right (**a**) and left (**b**) adrenal glands (arrow). Periaortic concentric soft tissue infiltration is visible (arrow). Axial CT-enhanced (**a**) and axial T1 post-gadolinium (**b**) images in a 71-year-old female also showing diffuse thickening of the left adrenal gland (arrow)

Retroperitoneal involvement frequently includes a bilateral, symmetric and diffuse thickening of the adrenal glands associated with infiltration of the adjacent fat (Figs. 5 and 7). Nevertheless, theses alterations rarely cause adrenal insufficiency [4, 15].

### Aorta and aortic branches

**Fig. 8:**
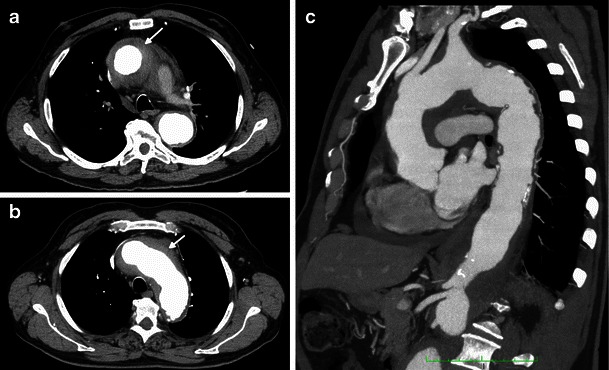
Axial and sagittal enhanced reformatted CT images of the thoracic aorta in a 73-year-old male showing periaortic infiltration (arrows) extending from the aortic root (**c**) to the descending aorta (**a**) and circumferentially involving the ascending aorta (**a**) and aortic arch (**b**)

**Fig. 9:**
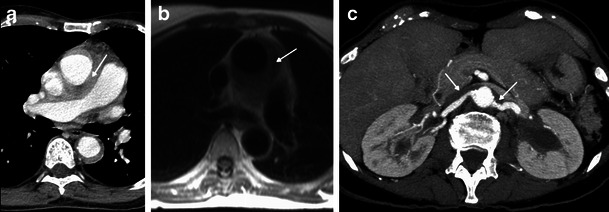
Axial CT-enhanced (**a**) and axial HASTE MR (**b**) images of the mediastinum and axial MIP CT (**c**) image of the upper abdomen in a 73-year-old male. There is periaortic and mediastinal soft tissue infiltration (arrow) and also infiltration of the origin of the renal arteries (arrows) and of the left renal sinus, with mild hydronephrosis (arrow)

**Fig. 10:**
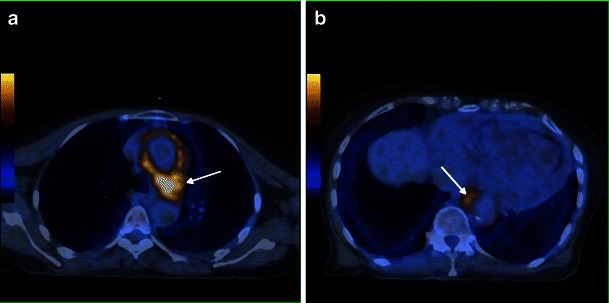
Axial FDG-PET/CT fusion images in a 71-year-old female, in the upper (**a**) and mid (**b**) portion of the thorax, showing elevated metabolic activity involving the aortic arch (arrow) and descending aorta (arrow)

The aorta is the vascular structure more commonly affected by this disease, and its involvement when diffuse looks like a regular circumferential periaortic infiltration, giving the appearance of a “coated aorta” [16]. However, the aorta may only be affected in a specific segment, symmetrically or asymmetrically [4]. This infiltration is preferentially peri-adventitial rather than parietal (Figs. 8, 9 and 10) [17] and may extend to the aortic branches (Fig. 9c), involving ostial segments of the supra-aortic, intercostal and coronary arteries [16] (Fig. 13) in the thorax and celiac trunk, renal and mesenteric arteries in the abdomen. This ostial extension, when severe, may cause arterial stenosis/occlusion, and consequently ischaemic changes in the respective organs [7] responsible for the symptoms—cerebral ischaemic events, arterial hypertension, cardiac insufficiency and abdominal angina. This infiltration has the same density and signal as that observed in the perirenal location: hypoattenuated on CT and isointense to muscle on T1 and T2 MR imaging, with weak contrast enhancement (Figs. 8, 9 and 10). On FDG PET/CT, it also has hypermetabolic activity (Fig. 10) [12].

Takayasu’s disease and retroperitoneal fibrosis constitute the main differential diagnosis. Takayasu’s disease differs from ECD by affecting the entire arterial wall, causing concentric thickening and conditioning thrombosis, stenosis and occlusion, vessel ectasia, aneurysms and ulcers [31].

### Heart and Pericardium

**Fig. 11:**
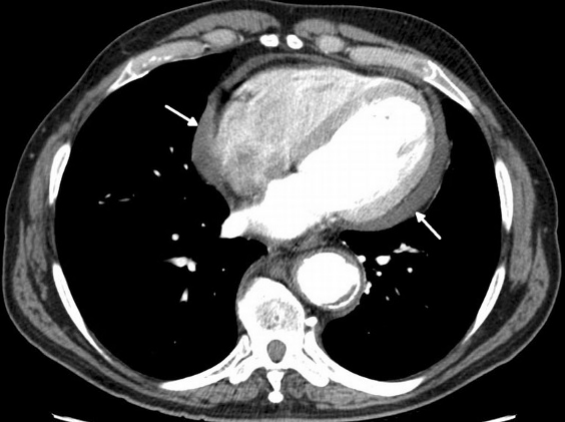
Axial enhanced CT images of the thorax in a 73-year-old male showing hypodense soft tissue infiltration of the pericardium (arrows)

**Fig. 12:**
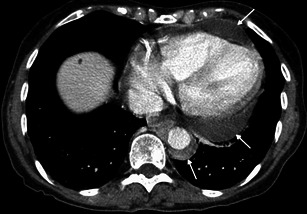
Axial enhanced CT images of the thorax in a 71-year-old female showing pericardial fluid (arrow) and periaortic infiltration (arrows)

**Fig. 13:**
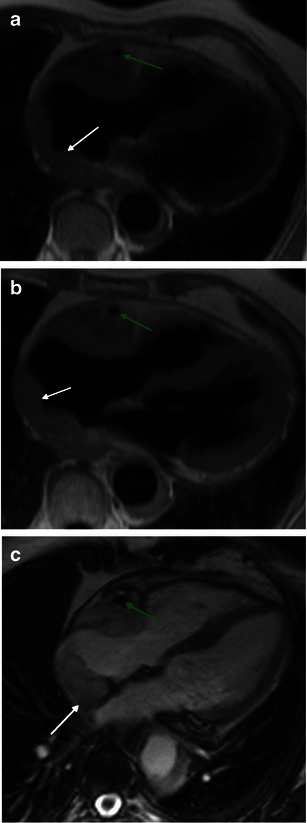
Axial HASTE (**a**), T1 (**b**) and four-chamber SSFP (**c**) cardiac MR images in a 71-year-old female, showing hypointense soft tissue infiltration of the right atrium and right atrio-ventricular sulcus (white arrrow), periarterial infiltration of the right coronary artery (green arrow) and periaortic infiltration (white arrow)

**Fig. 14:**
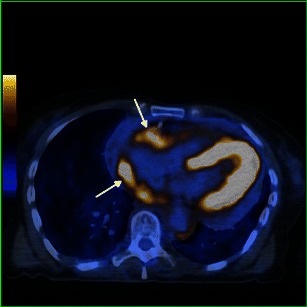
Axial FDG-PET/CT fusion images in a 71-year-old female, showing pericardial and epicardial thickening, with elevated metabolic activity, namely in the right atrium and atrio-ventricular sulcus (arrows)

Heart involvement occurs in 75 % of patients, may be symptomatic or not [18], and, in most cases, presents with ECG and imaging alterations [5]. It is very important to check the cardiovascular system in these patients because it carries the worst prognosis, with death occurring in nearly 60 % because of complications, namely severe arrhythmias, cardiomyopathy, myocardial infarction or valve insufficiency [5, 19]. Pericardium is the structure most commonly affected, presenting with smooth soft tissue thickening or effusion (Figs. 11 and 12) and sometimes leading to cardiac tamponade, which is fatal if not treated. Infiltration of the myocardium shows a predilection for right heart cavities, specifically the right atrium [5] and auriculoventricular sulcus [4], presenting frequently in the first location as a mural mass and simulating a tumoral—“pseudo-tumoral”—infiltration (Fig. 13). FDG PET/CT shows abnormal FDG accumulation in a pseudo-tumoral formation in the right atrium with thickened interatrial septum and involvement of the pericardium (Fig. 14).

### Lung parenchyma and pleura

High-resolution (HR) CT scans of the chest reveal involvement of the lung parenchyma and the pleura in 40 to 50 % of cases [20], but these alterations generally do not substantially affect the disease prognosis [4]. Patients are frequently asymptomatic or have some dyspnoea or dry cough and only rarely progress to respiratory insufficiency [4].

**Fig. 15:**
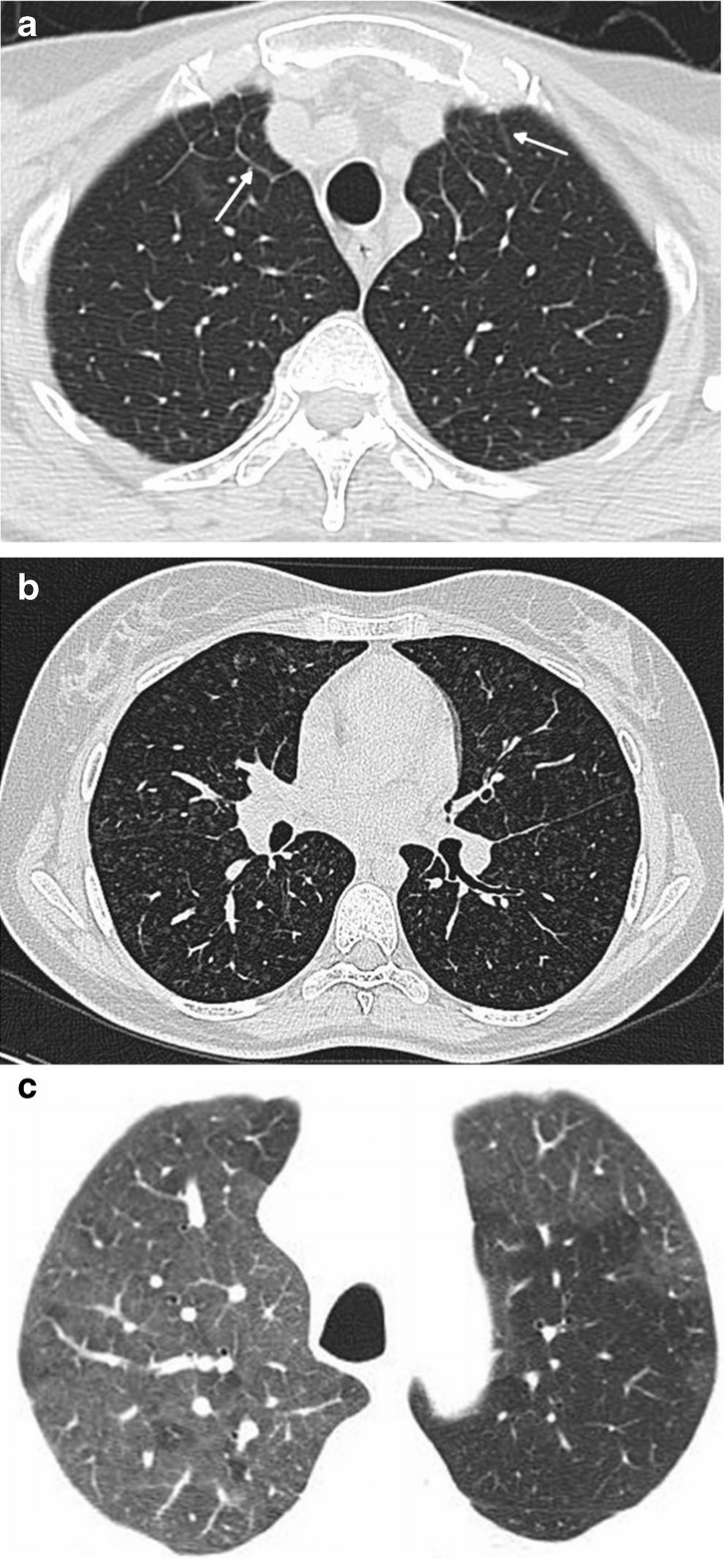
Several patterns of lung infiltration in high-resolution CT scans. **a** A 73-year-old male with bilateral smooth thickening of the interlobular septa at the lung apices (arrows); **b** a 39-year-old female with small centrilobular nodules at the mid-portion of the lungs; **c** a 71-year-old female with patchy areas of ground-glass attenuation at the lung apices

Pulmonary involvement in ECD has unspecific features but is very suggestive of the diagnosis in the appropriate context: smooth thickening of the pleura and fissures, usually bilateral and relatively symmetric ± pleural effusion, smooth septal thickening (Fig. 15a), small centrilobular nodules (Fig. 15b), parenchymal consolidation, cystic lesions and mosaic ground-glass opacities (Fig. 15c) [4, 21].

Initial pulmonary Langerhans cell histiocytosis may be difficult to differentiate from ECD because of the presence of septal thickening and nodules. Evidence of bizarrely shaped cysts and the typical evolution and distribution of LCH, from reticulo-nodular opacities to cysts, which tend to be most numerous in the upper and middle lung zones and spare the lung bases, is key to the differential diagnosis [32].

Cardiogenic interstitial oedema, sarcoidosis and pulmonary venoocclusive disease, other causes of septal thickening, must be differentiated from ECD. In cardiogenic interstitial oedema, although septal thickening may be smooth, centrilobular nodular opacities are unusual. In pulmonary sarcoidosis, septal thickening is usually nodular and is characteristic of the presence of adenopathy. In pulmonary venooclusive disease, there is similar smooth septal thickening, associated however with signs of pulmonary hypertension, such as enlargement of the central pulmonary arteries [33].

### Unusual localisations

Involvement of the breast [22], skeletal muscle [6, 22], liver [23], biliary ducts [24], mesentery [25], gastrointestinal tract [26] and testes [27] is extremely rare in ECD and only described in clinical reports.

## Making the diagnosis of ECD

The diagnosis of ECD can be made with near certainty according to its characteristic imaging findings. The bilateral diffuse or patchy symmetric osteosclerosis of the major long bones, with relative epiphyseal sparing, associated with perirenal disease or aortic perivascular thickening, is virtually pathognomonic for ECD. However, the definitive diagnosis is made according to its distinct histological findings [4]. Biopsy samples from the involved organs, preferentially bony and retroperitoneal infiltration [4], will reveal diffuse xanthogranulomatous infiltration with foamy histiocytes, inflammatory and Touton giant cells surrounded by fibrosis. Immunohistochemistry of the biopsy samples confirms the monocyte/macrophage lineage of the histiocytes by their expression of CD68, lysozymes and CD4 and distinguishes them from the Langerhans cell type by the lack of the CD1a and S100 protein markers. Electron microscopic findings strengthen the immunostaining results and confirm the non-Langerhans cell type by lack of Birbeck granules in their ultrastruture [4, 5].

## Role of imaging

Since the outcome of ECD is related to the visceral involvement, particularly cardiovascular and pulmonary, following the establishment of the diagnosis, it is essential to assess the presence of visceral involvement using CT, MRI and/or FDG-PET/CT.

Radiographic evaluation of the long bones is essential for the workup of this condition [9]. Bone scintigraphy allows a global evaluation of skeletal abnormalities, which helps to detect radiographically and clinically silent bone involvement [34].

CT and HRCT are useful for better detection and characterisation of lung, pleural, aortic and skeletal abnormalities. CT is also the technique more commonly used for image-guided biopsy [21].

MR imaging is the best technique for the detection and characterisation of cardiac and pericardial abnormalities [19]. MR imaging of the abdomen remains an alternative in patients with contraindications to iodinated IV contrast media.

Finally, FDG PET/CT has the main benefit of simultaneously evaluating the extent of the skeletal and extraskeletal disease, detecting occult visceral and vascular involvement and following its evolution and the response to treatment [4, 11].

## Conclusions

The manifestations in Erdheim-Chester disease are diverse and nonspecific, and patients may go years before they get a correct diagnosis.

Symmetric osteosclerosis of the long bones involving the metaphyses and diaphyses but sparing the epiphyses is the most characteristic finding. The disease may also affect the central nervous system, heart, pericardium, lungs and kidneys, retroperitoneal and retro-orbital tissue.

The number of new cases has dramatically increased over the past years because of the better recognition of this condition. The natural evolution is variable but the spontaneous prognosis is severe. A good knowledge of its specific imaging features seems to be crucial for early management and improved prognosis.

## Abbreviations

ECD: Erdheim-Chester disease
FDG: 2-[Fluorine-18] fluoro-2-deoxy-d-glucose
PET: positron emission tomography
LCH: Langerhans cell histiocytosis
